# Mindfulness-Based Virtual Reality Intervention for Children and Young Adults with Inflammatory Bowel Disease: A Pilot Feasibility and Acceptability Study

**DOI:** 10.3390/children8050368

**Published:** 2021-05-05

**Authors:** Anava A. Wren, Nicole Neiman, Thomas J. Caruso, Samuel Rodriguez, Katherine Taylor, Martine Madill, Hal Rives, Linda Nguyen

**Affiliations:** 1 Division of Gastroenterology, Hepatology and Nutrition, Department of Pediatrics, Stanford University School of Medicine, Stanford, CA 94304, USA; awren2@stanford.edu; 2 Division of Pediatric Anesthesiology, Department of Anesthesiology, Perioperative, and Pain Medicine, Stanford University School of Medicine, Stanford, CA 94304, USA; tjcaruso@stanford.edu (T.J.C.); sr1@stanford.edu (S.R.); kgtaylo@umich.edu (K.T.); martinemadill@gmail.com (M.M.); hdr34@cornell.edu (H.R.); 3 Division of Gastroenterology and Hepatology, Department of Medicine, Stanford University School of Medicine, Redwood City, CA 94063, USA; nguyenlb@stanford.edu

**Keywords:** Inflammatory Bowel Disease, Virtual Reality, mindfulness, Mindfulness-Based Interventions

## Abstract

The aim of this pilot study was to assess: (1) the feasibility and acceptability of a Mindfulness-Based Virtual Reality (MBVR) intervention among children and young adults with Inflammatory Bowel Disease (IBD), and (2) the preliminary efficacy of MBVR on key psychological (anxiety) and physical (pain) outcomes. Participants were 62 children to young adults with IBD (M = 15.6 years; 69.4% Crohn’s disease; 58% male) recruited from an outpatient pediatric IBD clinic. Participants completed a baseline assessment, underwent the 6-min MBVR intervention, completed a post-intervention assessment and study satisfaction survey, and provided qualitative feedback. Results suggest strong feasibility and acceptability. Participants reported high levels of satisfaction with MBVR including high levels of enjoyment (M = 4.38; range 1–5) and relaxation (M = 4.35; range 1–5). Qualitative data revealed several key themes including participants interest in using MBVR in IBD medical settings (e.g., hospitalizations, IBD procedures, IBD treatments), as well as in their daily lives to support stress and symptom management. Preliminary analyses demonstrated improvements in anxiety (*t* = 4.79, *p* = 0.001) and pain (*t* = 3.72, *p* < 0.001) following MBVR. These findings provide initial support for the feasibility and acceptability of MBVR among children and young adults with IBD. Results also suggest MBVR may improve key IBD outcomes (e.g., anxiety, pain) and highlight the importance of conducting a randomized controlled trial and more rigorous research to determine intervention efficacy.

## 1. Introduction

Inflammatory Bowel Disease (IBD) is a chronic autoinflammatory disease affecting a growing number of children and young adults in the United States [1]. Approximately 25% of individuals with IBD are diagnosed before 20 years old [2], with peak onset in adolescence [3]. Recently, the prevalence of pediatric IBD (2–17 years old) was shown to be 77 per 100,000 children in the United States, demonstrating a 133% increase from 2007 to 2016 [4]. Many aspects of living with IBD can lead to increased psychosocial stress for children and young adults such as active flares, missed school for regular doctor appointments, and frequent phlebotomy, procedures, and treatments. While varying individual, family, and social factors can influence how an individual adjusts and copes with IBD, children to young adults with IBD experience higher levels of psychological distress (e.g., anxiety, depression) [5,6]. A recent systematic review and meta-analysis described prevalence estimates of anxiety and depression in children with IBD; pooled prevalence was 16.4% for anxiety symptoms, 4.2% for anxiety disorders, 15% for depressive symptoms, and 3.4% for depressive disorders [7]. Children with IBD have also been shown to have higher rates of internalizing disorders compared to children with other chronic illnesses [5]. Of note, psychological distress has been associated with disease activity and shown to be a predictor of flares and other negative health outcomes [8,9,10]. Poor health outcomes can in turn increase distress [9,11], which may lead to a cycle involving the brain-gut axis, highlighting the need for behavioral interventions that can improve biopsychosocial processes and support effective multidisciplinary care for pediatric IBD.

Mindfulness-Based Interventions (MBIs) have been shown to be efficacious in improving biopsychosocial outcomes such as pain, inflammation, and emotional distress in a range of pediatric and adult chronic illness/pain populations [12,13,14,15]. Across these MBIs, treatments are focused on teaching mindfulness—the ability to be aware of the present moment and one’s thoughts, emotions, and body sensations in a purposeful, non-judgmental manner [16]. Mindfulness is often taught through breathing and meditation exercises that focus on bringing one’s attention to the present. Cultivating this type of focus and non-judgmental, compassionate awareness can buffer individuals from ruminating on negative thoughts (e.g., anxiety about IBD) or bothersome physical sensations (e.g., pain), as well as lead to changes in the autonomic nervous system and immune system [17,18]. 

The majority of MBIs for individuals with IBD have been conducted in adult populations. These studies have shown promising results including significant improvements in anxiety, depression, and quality of life, as well as initial support for improvements in inflammatory markers [17,19]. Two studies have explored group-based MBIs in pediatric IBD samples. One study demonstrated feasibility of an 8-week MBI group and improvements in emotional functioning [20]. Another 8-week MBI group (grounded in mindfulness-based cognitive therapy) showed positive qualitative findings including children with IBD feeling more connected to others and more accepting of IBD [21]. These preliminary findings, coupled with the adult IBD research, highlight that MBIs may have the potential to improve biopsychosocial outcomes among pediatric IBD populations. 

To date, MBIs in pediatric and adult IBD populations have been conducted in longer-term training models (e.g., 8-weeks) which is representative of the broader mindfulness literature [22]. More intensive MBIs provide more exposure to mindfulness and can support longer-term mindfulness practice which has been shown to be associated with positive outcomes such as changes in neural networks involved in emotion regulation [23]. However, the more intensive treatment model and time commitment can be a barrier to many who otherwise might benefit from MBIs and can be challenging to administer in many clinical settings [24]. Thus, there has been growing interest in whether brief MBIs can impact health outcomes. While recent reviews have demonstrated mixed results and more research is needed, studies have provided some preliminary support for brief MBIs (one session over 5 min) impacting acute pain and psychological outcomes including anxiety in healthy and clinical populations [24,25,26]. Brief MBIs may hold particular promise for pediatric populations as MBIs for children are often adapted and shortened to ensure developmental appropriateness (e.g., attention span and cognitive abilities) [27]. Exploring whether brief MBIs could support improvements in key psychological (e.g., anxiety) and physical (e.g., pain) outcomes in children and young adults with IBD is an important area of study, as they could be easily delivered in medical settings and enhance multidisciplinary pediatric IBD care. Brief MBIs could support multidisciplinary IBD teams improve clinical care as they have the potential to provide acute symptom management across a variety of IBD medical settings and facilitate adjustment to IBD. Overall, brief MBIs may be able to improve the physical and psychological wellbeing of children and young adults with IBD and should be a prioritized area of research within pediatric IBD.

When considering delivery methods for brief MBIs among pediatric IBD populations, Virtual Reality (VR) platforms have great potential. VR’s increasing availability in children’s hospitals, high acceptability among children, and immersive nature [28,29,30] all suggest it could provide an excellent platform to deliver engaging MBIs. Past research in adult populations has demonstrated that MBIs delivered via VR can help actively capture a person’s attention and support their sense of presence in the mindfulness experience (via 3D computer generated environment) [31,32,33]. Studies exploring brief MBIs in VR among adult samples of novice and experienced meditators have demonstrated positive findings such as increases in state mindfulness and psychological wellbeing [32,34,35]. A recent study suggests that a brief MBI delivered by VR reduced anxiety among children who were homeless [36]. No published studies to date have been conducted in clinical pediatric populations. 

Thus, the purpose of this study was to: (1) Assess the feasibility and acceptability of a brief mindfulness-based Virtual Reality (MBVR) intervention among children and young adults with IBD in an outpatient medical setting (pediatric IBD clinic); and (2) Assess the preliminary efficacy of MBVR on key psychological (i.e., anxiety) and physical (i.e., pain) outcomes. 

## 2. Materials and Methods

### 2.1. Participants

The participants in this study were 62 patients with IBD recruited from Stanford Children’s Inflammatory Bowel Disease Center from 06/2019 through 11/2019. To be in enrolled in the study, participants had to meet the following criteria: (1) have a diagnosis of Ulcerative Colitis or Crohn’s disease, (2) be at least 10 years of age, (3) be able to speak, understand, and read English, (4) have no history of cognitive impairment, developmental delay, seizure disorders, severe visual impairment, or motion sickness with VR. Patients were required to be at least 10 years of age for developmental considerations (e.g., attention span, language and cognitive abilities) and due to past research that has supported children 10 years of age or older being able to engage in mindful attention exercises [37]. Additionally, as this was a pilot study focused on the feasibility and acceptability of MBVR, a wide participant age range was targeted to deliver the intervention to the patient population served in our pediatric IBD clinic (i.e., children to young adults). 

**Figure 1:**
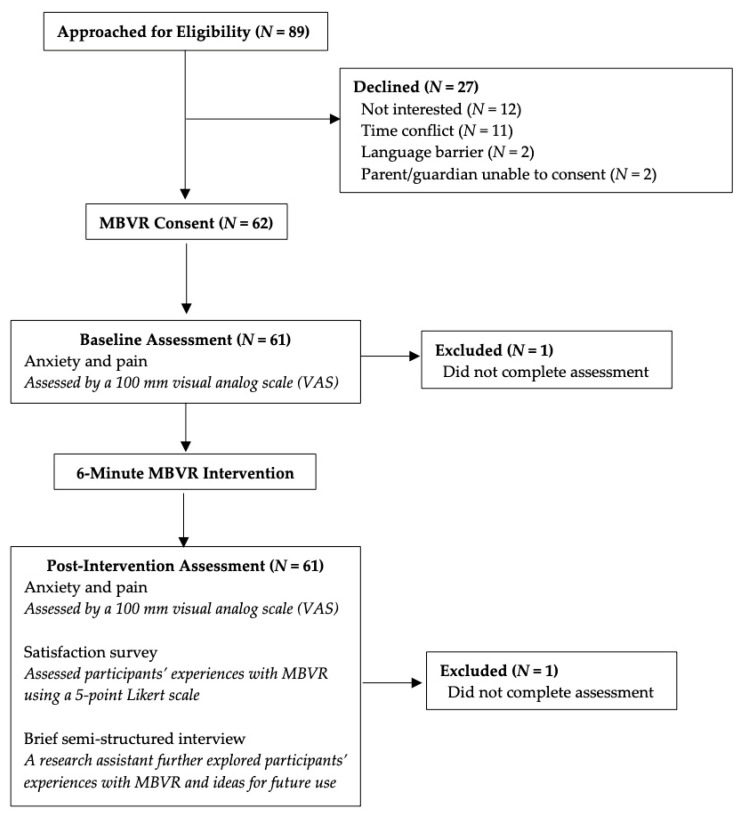
Study flow.

Of the 89 participants invited to this study, 62 provided informed consent. The two most common reasons for patients not consenting were lack of time and interest. See Figure 1 for study recruitment and flow. All procedures and protocols were approved by the Stanford University Institutional Review Board.

### 2.2. Procedures

All eligible patients presenting to the IBD Center were provided with an overview of the study. Research assistants followed a recruitment script that briefly introduced the study and explored patients familiarity with mindfulness and VR. Standardized talking points were followed to provide patients and families with more information about mindfulness (definition and a brief overview of how MBIs have been shown to improve health outcomes in a range of patient populations); how mindfulness might be able to support children with IBD; and an overview of the MBVR intervention (guided mindfulness practice paired with visual effects in VR). If interested, patients and parents completed a written assent and consent, respectively. Depending on clinic flow, participants completed the study either before or after their clinic appointment. Participants were brought into a private clinic room and completed a baseline assessment, specifically rating their current level of anxiety and pain on a 100 mm visual analog scale (VAS). Next, participants were fitted with a customized Samsung Gear VR headset (Samsung Electronics, Suwon, South Korea) equipped with a Samsung Galaxy S8 smartphone. The headsets were customized to have wipeable face pads, 3D printed straps, and ratchets, so surfaces could be sanitized with hospital-approved sanitation methods between participants. Research assistants oriented participants in the VR, so they could engage in the MBVR experience supine or sitting upright. Participants then completed the 6-min MBVR intervention; all research assistants exited the clinic room during the intervention. After completing the MBVR, participants completed a post-intervention assessment, rating their levels of anxiety and pain on a 100 mm VAS, and completed a study satisfaction survey. Qualitative feedback about participants’ experiences with MBVR was obtained through a brief semi-structured interview with a research assistant. The purpose of the qualitative interview was to build upon the satisfaction survey and learn more about participants’ experiences with MBVR and their ideas for areas of future use. The whole study was completed in a single visit with a research assistant coordinating all aspects of the study visit. The study took between 15 to 30 min depending on how long the consent process and interview lasted. The majority of participants finished the brief semi-structured interview within five minutes.

### 2.3. Intervention

**Figure 2:**
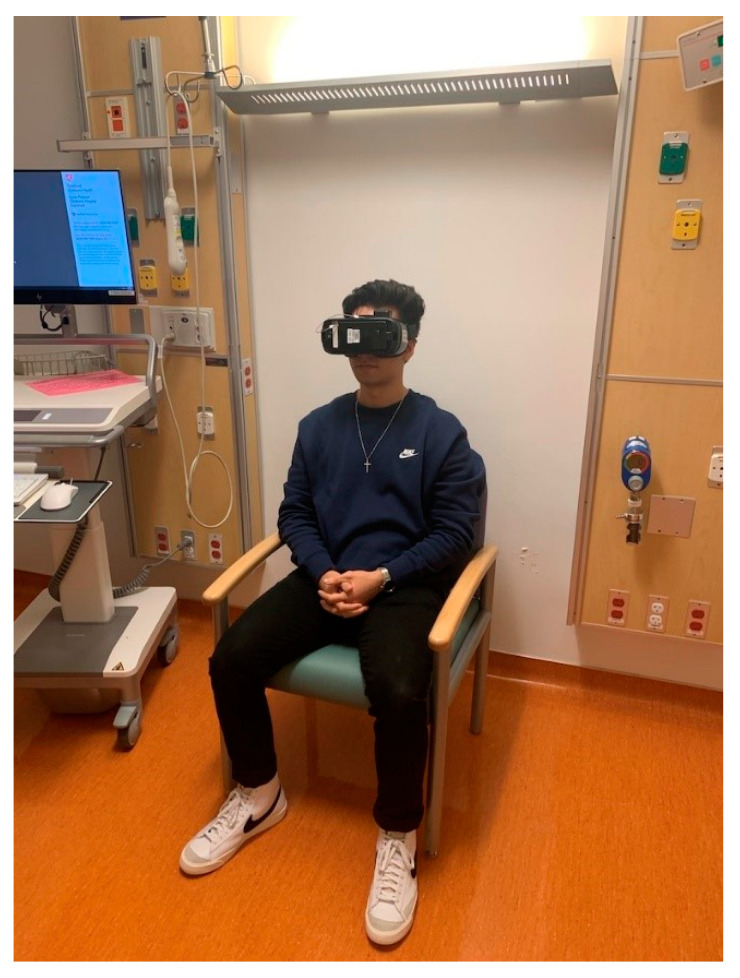
Mindfulness-Based Virtual Reality (MBVR) Application and Set-Up. The MBVR application was pre-loaded on a Samsung Gear VR headset equipped with a Samsung Galaxy S8 smartphone. Participants could engage with the MBVR experience sitting upright in a chair or supine in a medical exam bed.

The MBVR intervention was delivered using a VR headset that was pre-loaded with a 6-min first-person point of view MBVR application, “MediMindfulness-Transitions,” developed by the Stanford Chariot Program (Figure 2). In developing the MBVR application, the Stanford Chariot Program aimed to create an application that would deliver a brief MBI between 5–10 min to support implementation into a busy clinical setting. The length of this intervention was guided by past research that supports the use of shorter MBIs among children due to developmental considerations such as attention span [27], as well as studies that support the effectiveness of brief MBIs of at least five minutes [24,26]. The same MBVR application was delivered to all study participants and no modifications were made across age. The MBVR application was created to be accessible to children through young adults.

Creation of the MBVR application was a collaborative effort between core members of Stanford’s Chariot Program—a pediatric IBD psychologist with extensive clinical and research experience creating and delivering MBIs across pediatric and adult chronic illness and chronic pain populations; a gastroenterologist; and two pediatric pain anesthesiologists who are innovators in using technology such as VR to support pediatric populations with symptom management (e.g., anxiety, pain). In the MBVR application, a guided mindfulness voiceover was synched with visual effects in VR created specifically for this study. While the guided mindfulness practice was written for this study, it was grounded in past mindfulness research and theory [38]. The mindfulness practice was centered around cultivating focused-attention and present moment awareness which is supported by past research on brief MBIs (i.e., short mindfulness protocols that teach one central component of mindfulness) [26,32]. Specifically, the mindfulness practice focused on bringing participants’ attention and awareness to their breath and natural virtual environments (i.e., waterfall in meadow, northern lights). 

**Figure 3:**
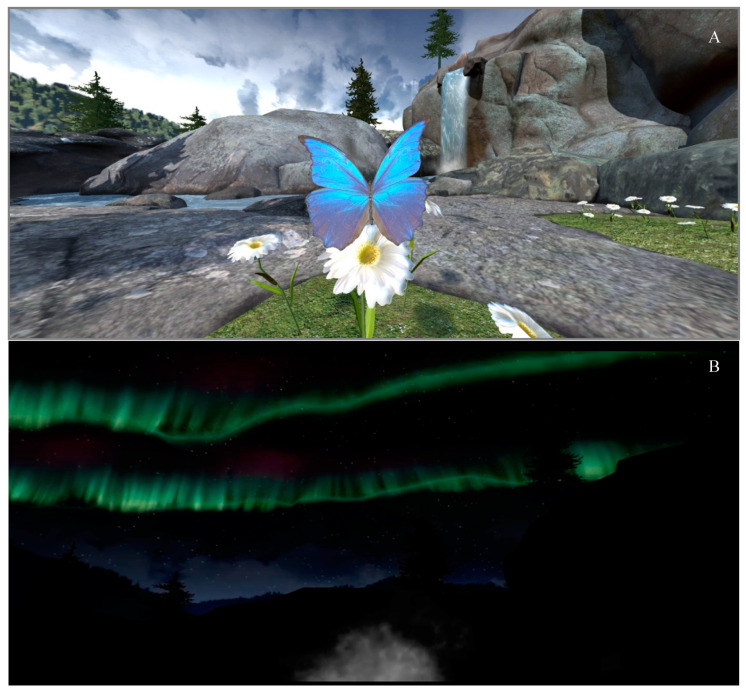
MBVR Experience. (**A**) This panel shows the first scene in MBVR (i.e., peaceful meadow with a waterfall) and the butterfly that supports participants pace their breath. (**B**) This panel displays the second scene in MBVR (i.e., night sky with northern lights) and the breath mist that supports participants pace their breath.

The MBVR application began with a female narrator briefly discussing mindfulness, specifically what it is, how everyone has experienced mindfulness or present-moment awareness (providing examples such as being fully focused on an activity such as playing an instrument or sport), and how mindfulness can support them in their daily lives (e.g., times of discomfort or stress). The narrator then provided practical suggestions before starting such as bringing one’s full attention to the mindfulness practice and what to do if their minds wandered. Then, the guided mindfulness practice began with the narrator using invitational language to guide participants’ attention to their breath and physical sensations in their body, grounding the practice with mindful breathing. Participants were then invited to shift their focus to a peaceful meadow with a waterfall (Figure 3) and explore the new environment using their senses (e.g., listen to the water falling, watch the clouds moving). Later, they were invited to focus their attention on a butterfly in the meadow. Participants were encouraged to observe the butterfly’s movements and then synch their breath with the butterfly, using the butterfly’s movements (floating up and down) to pace and slow their breath. Attention was also brought to their own breath cycle during this exercise. About halfway through the MBVR experience, participants were invited to notice how the meadow was slowly becoming darker, dusk changing to nighttime. Their awareness was then shifted to the night sky and northern lights, encouraging participants to use their senses to observe the changing landscape. Participants’ were again invited to focus their attention on their breath and how it naturally synched with objects in their environment (e.g., northern lights moving in and out, mist of breath moving in and out) (Figure 3). The narrator also allowed for brief pauses and silence to practice the mindful awareness that was being cultivated in the mindfulness experience.

During the guided mindfulness practice, the narrator also offered suggestions about how mindfulness could lead to increased relaxation and comfort. For example, the narrator shared that when participants shifted their full awareness to one thing such as their breath, they allowed themselves to let go of any thoughts, worries, or discomfort and increase comfort and relaxation in their body. At the end, participants received encouragement and guidance on how to continue their mindfulness practice in their daily lives. 

### 2.4. Measures

*Anxiety.* Anxiety was measured with a 0–100 mm visual analog scale (VAS). Participants were asked to indicate their level of anxiety by marking along a 100 mm line (0 = No Anxiety, 100 = Worst Anxiety). Anxiety VAS scales are commonly used in research examining anxiety and studies have supported their reliability, validity, and specificity [39].

*Pain.* Pain intensity was measured with a 0–100 mm visual analog scale (VAS). Participants were asked to indicate their level of pain intensity by marking along a 100 mm line (0 = No Pain, 100 = Worst Pain). Pain VAS scales are commonly used in research examining pain and studies have supported their reliability, validity, and specificity [40,41].

*Demographics and Medical Variables.* Demographic and medical variables were obtained from electronic medical records.

*Feasibility and Acceptability.* A satisfaction questionnaire was created for this study and completed by participants after the MBVR intervention. Using a 5-point Likert scale [1 (poor/not at all) to 5 (excellent/ideal)], questions assessed participants’ feelings of enjoyment and relaxation after MBVR, as well as their feelings regarding the length of MBVR and interest in using MBVR at home. Participants were also provided with space for additional comments and suggestions about their experience with MBVR. Satisfaction with MBVR was further assessed through a brief semi-structured interview with a research assistant following completion of post-intervention study questionnaires. This interview focused on further exploring participants’ experiences with MBVR and ideas for future use. Participants were asked to: (1) Discuss their experience with MBVR (positives and negatives/what they would recommend changing), and (2) discuss their ideas about how to use MBVR in the future. Participant accrual (at least a 60% participation rate), attrition (at least 80% of consented participants completing the study protocol), and adherence (proportion of participants successfully completing all assessments, with at least 75% completion rates serving as a benchmark) were included as an additional component of overall feasibility.

### 2.5. Data Analysis

To answer the first aim of the study, feasibility and acceptability were defined in terms of participants’ ratings on the satisfaction questionnaire in addition to participant accrual, attrition, and adherence. Given the exploratory nature of this study, descriptive and summary statistics were used to assess satisfaction ratings and feasibility of the intervention. Qualitative data was also obtained pertaining to participants’ experiences with MBVR and areas for future use. To answer the second aim of the study, preliminary efficacy was assessed via a series of two-tailed paired samples t-tests to assess changes in participants’ anxiety and pain pre-post MBVR. Data was analyzed using SPSS version 26.

Exploratory analyses were conducted to better understand the impact of age (<18 years old & ≥18 years old) and IBD diagnosis (Crohn’s & Ulcerative Colitis) on study outcomes. To assess the impact of age on study feasibility and efficacy, separate summary statistics and two-tailed paired samples t-tests were conducted for both age groups (<18 years old & ≥18 years old). Further, analyses of covariance (ANCOVA) were used to assess whether levels of anxiety and pain differed by IBD diagnosis (i.e., Crohn’s vs. Ulcerative Colitis) following MBVR (controlling for respective symptom levels at baseline).

## 3. Results

### 3.1. Descriptive Statistics

Consenting participants included 62 individuals between the ages of 10 and 25 (M = 15.6 years; SD = 3.29 years). In this sample, 69.4% had Crohn’s disease and 30.6% had Ulcerative Colitis; 58% identified their gender as male; 58% identified as Caucasian, 17.7% identified as Asian/South Asian, 1.6% identified as African American, 22.6% identified as Other; 92% identified as non-Hispanic. 

### 3.2. Feasibility and Acceptability

Among eligible patients approached for the study (*N* = 89), 62 consented to participate (70% participation accrual rate). Among consented participants, all completed the intervention (100% retention). Adherence to assessments was 98%. Across pre/post assessments, two assessments were not completed due study assessment error (i.e., one participant did not complete part of the pre-intervention assessment which was not noticed until after the patient left clinic; one participant did not complete the post-intervention assessment which was not noticed until after the patient left clinic).

Overall, participants reported high levels of acceptability and satisfaction with MBVR (range: 1–5). The majority of participants reported high levels of enjoyment (M = 4.38; SD = 0.897) and relaxation (M = 4.35; SD = 0.833) following MBVR. About half of the participants reported they felt the length of the intervention was ideal (M = 4; SD = 1.13) and were interested in using MBVR again at home (M = 4.10; SD = 1.09). 

Qualitative data revealed several key themes when assessing participants’ experiences with MBVR and areas for future use. Participants reported they: (1) found MBVR enjoyable and relaxing; (2) would like to use MBVR in a variety of IBD clinical settings (e.g., inpatient hospitalizations, procedures, treatments, labs); (3) would like more access to MBVR in their daily lives (e.g., ability to purchase it and use it at home, school or work). Participants also provided user experience feedback regarding future use of MBVR. Table 1 shows a selection of participant responses when asked about their MBVR experiences and areas for future use. There were no reports of injury or safety concerns after MBVR though one participant endorsed feeling some dizziness during MBVR and another participant felt more anxious after MBVR. Both participants completed MBVR and provided this feedback post-intervention.

### 3.3. Preliminary Efficacy

Analyses were conducted to examine preliminary efficacy of MBVR. Participants reported a significant decrease in anxiety (16.61 ± 20.84 to 7.09 ± 13.36; *t* = 4.79, *p* = 0.001) and pain (9.97 ± 16.51 to 3.15 ± 6.38; *t* = 3.72, *p* < 0.001) post-intervention. 

### 3.4. Exploratory Analyses

Exploratory analyses were conducted to examine the impact of age (<18 years old, ≥18 years old) on study feasibility and efficacy. Both age groups reported high levels of acceptability and satisfaction with MBVR, similar to findings above with the full sample. Summary statistics were also similar between age groups (see Table 2). Among participants < 18 years old (*N* = 42), there was a significant decrease in anxiety (18.54 ± 23.47 to 7.38 ± 13.03; *t* = 4.30, *p* < 0.001) and pain (11.17 ± 17.51 to 3.10 ± 6.53; *t* = 3.43, *p* = 0.001) post-intervention. Among participants ≥ 18 years old (*N* = 19), there was a significant decrease in anxiety (12.11 ± 12.23 to 6.50 ± 14.53; *t* = 2.21, *p* = 0.41), but not in pain (7.22 ± 13.96 to 3.33 ± 6.33; *t* = 1.47, *p* = 0.16) post-intervention. 

When exploring differences in MBVR outcomes by diagnostic subgroup, the results suggest no differences between individuals with Crohn’s disease versus Ulcerative Colitis in pain intensity (F(1, 57) = 0.39, *p* > 0.05) or anxiety (F(1, 57) = 0.69, *p* > 0.05) following the intervention. 

## 4. Discussion

To our knowledge, this is the first pilot study investigating the use of a mindfulness-based Virtual Reality intervention among children and young adults with IBD. Results offer preliminary evidence that a brief MBVR intervention is feasible and acceptable, and can improve anxiety and pain levels in a pediatric IBD sample. 

When examining the study’s feasibility and acceptability, there was notable interest in the MBVR intervention among children and young adults with IBD, evidenced by the positive participant accrual rate (70%) and intervention retention (100%). Participant enjoyment of the intervention was also highly rated. Many participants indicated that they found the 6-min mindfulness experience in VR very accessible and engaging (e.g., the interactive mindful breathing exercises that used a floating butterfly and the northern lights to support paced breathing). Participants also expressed an interest in using MBVR at home to support their IBD health and stress related to other domains of their life (e.g., school, work, sports). These findings support past VR mindfulness research that suggests VR may offer a unique platform for MBIs as the technology can actively capture a person’s attention and support their sense of presence in mindfulness exercises, as well as lead to increased treatment adherence [31]. Thus, utilizing VR platforms to teach and practice brief mindfulness protocols could support past barriers in pediatric mindfulness research such as challenges focusing on longer mindfulness practices and engaging in home practice [21]. 

Additionally, study results provided preliminary support for MBVR improving anxiety and pain among children and young adults with IBD. Qualitative findings highlighted that participants found MBVR relaxing, calming, and soothing for their mind and body, leading to less anxiety, stress, and pain after the intervention. Participants also endorsed high levels of relaxation following MBVR. This was further supported by analyses showing statistically significant reductions in anxiety and pain levels in the full sample post-intervention. When performing exploratory analyses and examining outcomes by age group, results demonstrated that children < 18 old years (*N* = 42) had significant reductions in anxiety and pain levels post-intervention, while young adults ≥ 18 years old (*N* = 19) had significant reductions in anxiety but not pain levels post-intervention. Of note, the sample of young adults was smaller and had lower levels of baseline pain compared to the sample of children. This an interesting finding that requires further attention and study in a trial with a larger sample of young adults to determine whether this nonsignificant result holds or if MBVR can reduce pain levels among young adults. This result also highlights an important future research direction—exploring dose effects of MBVR by age to see if children benefit from shorter mindfulness experiences in MBVR and young adults benefit from slightly longer mindfulness experiences in MBVR (e.g., 10–15 min). 

Overall, these findings support past research that has shown MBIs can improve psychological distress and physical symptoms such as pain in pediatric and adult populations [13,14,15]. These are particularly promising findings as children and young adults with IBD are a high-risk population. Research has shown that children with IBD experience higher rates of psychiatric disorders compared to healthy children and those with other chronic illnesses [5,6], increasing rates of chronic abdominal pain and chronic opioid use [42,43,44], and lower quality of life [5,6], all of which can negatively impact emotional and physical functioning in adulthood. As a result, there has been an increased call for behavioral interventions to support psychological and physical wellbeing among children with IBD [6,45]. Given these positive preliminary findings, MBVR is a novel behavioral intervention that should be further explored in larger randomized controlled trials (RCTs).

Another noteworthy finding was participants’ interest in using MBVR in a range of IBD medical settings. One of the primary themes that emerged from the qualitative analyses was that youth with IBD expressed a desire to access MBVR in outpatient clinic settings as well as during procedures (e.g., colonoscopy, endoscopy, MRI/MRE), treatments (e.g., receiving an infusion or injection), lab visits (e.g., blood draws), and generally during hospitalizations. Children to young adults shared that they thought MBVR could support anxiety and pain management across many IBD settings. This is an incredibly important area of future research, as administering a brief MBVR intervention in a range of inpatient and outpatient medical settings could lead to increased accessibility to Mindfulness-Based Interventions (MBIs) among children and young adults with IBD. To date, most MBIs have been delivered in person by mental health providers, or in individual and group therapy settings. However, by using VR, brief MBIs could be delivered to children by a variety of providers on multidisciplinary IBD teams (e.g., IBD gastroenterologist on an inpatient unit, IBD nurse before a colonoscopy, IBD medical assistant before a lab visit). Providers would need to be trained and provided with information and talking points when delivering the intervention to families (e.g., a script that introduces mindfulness/MBVR and highlights potential benefits of use); that said, the majority of psychoeducation about mindfulness could occur in VR by a guided narrator before the mindfulness exercise. Increased access to brief MBIs that target biopsychosocial processes could support multidisciplinary IBD teams in providing children and young adults with improved symptom management (e.g., anxiety, pain) in medical settings and provide more holistic IBD care.

Additionally, as increasing numbers of IBD clinics occur over telemedicine, behavioral health interventions that can be delivered remotely are of increased importance. Fortunately, VR allows for more remote teaching and exposure to behavioral interventions such as MBIs and can be easily used in a home setting. Past research has supported VR use at home with a variety of devices including self-guided app-based VR interventions that use smartphones and low-cost cardboard VR goggles ($10) [46].These studies demonstrate how VR interventions can be easily delivered at home and improve accessibility to behavioral interventions which has been a past barrier to in-person mindfulness interventions among children with IBD (e.g., recruitment and retention challenges due to travel time to medical centers and duration of intervention) [20,21]. Participants in this study expressed a desire to use MBVR at home to support stress and symptom management, further indicating this could be a valuable future direction in pediatric IBD research. Specifically, it will be important to explore the feasibility and efficacy of a self-guided app-based MBVR intervention designed for a home setting, as well as assess longer-term use of MBVR (e.g., 4 weeks) on key IBD outcomes. 

Considering how multidisciplinary IBD teams could “prescribe” longer-term MBVR practice for improved symptom management and psychological wellbeing is a particularly interesting clinical question and area of research. For instance, exploring how gastroenterologists, mental health providers, and other IBD support staff could prescribe MBVR to support stress and anxiety management before procedures (e.g., practice daily for two weeks before a colonoscopy or surgery) or when someone has persistent pain concerns (e.g., practice daily in between monthly infusions) would be a valuable area to explore. Interestingly, examining ongoing use of MBVR protocols has been highlighted as a key future direction in better understanding how VR can support mindfulness practice [32]. Some researchers have begun to design and explore how VR can support longer-term mindfulness interventions such as an 8-week Mindfulness Based Stress Reduction (MBSR) protocol [47] though additional research is needed on intervention efficacy. 

While the study’s preliminary results are promising, it is important to consider the current findings in the context of several limitations. In particular, the study is limited by its lack of control group and random assignment. As a result, it is difficult to determine whether the positive preliminary efficacy findings are due to the intervention or other factors (e.g., patient expectancies, positive attention during the intervention, application features). Additionally, it will be important to assess whether MBVR is superior to other VR interventions used clinically (e.g., those that are focused on active distraction). Second, the study included a diverse array of youth with IBD who varied in terms of presenting clinical symptoms, disease activity, and treatment regimen. While recruiting a heterogeneous sample is not inherently a study limitation, the smaller sample size did not allow for additional analyses that could have examined specific intervention differences between the various groups of patients (e.g., active disease versus remission; presenting with pain versus no pain; met criteria for a psychiatric disorder versus no psychiatric disorder). To further assess the external validity of the study, future studies should recruit a larger sample and conduct appropriate stratified analyses across groups to assess the efficacy of MBVR among sub-populations of youth with IBD. While this study demonstrated statistically significant reductions in anxiety and pain pre- to post-intervention, these changes were not clinically significant. Exploring the efficacy of MBVR in sub-populations of youth with IBD presenting with active flares, persistent pain, and psychiatric disorders or clinically elevated levels of psychological distress (e.g., anxiety) could support further exploration into whether MBVR can lead to clinically significant reductions in key psychological and physical outcomes. Third, there was the potential for study bias, including social desirability bias, as research assistants conducted all parts of the study visit (i.e., consent, intervention delivery, assessments). Having the same research assistant conduct the intervention delivery and assessments could impact participants’ survey responses and qualitative feedback. Future RCTs should implement blinding techniques to the greatest extent possible or have separate research assistants deliver the intervention and conduct assessments to reduce the potential for bias. Future studies should also ensure more standardized intervention delivery. In the current pilot feasibility study, MBVR was delivered either before or after IBD clinic appointments, based on clinic flow and participant availability. It is possible that baseline anxiety scores were impacted by appointment-related stress for those who received the intervention before their clinic appointment. In future study procedures, all participants should receive MBVR at the same time during clinic appointments. Fourth, all data was self-report. The absence of objective biophysiological data (e.g., heart-rate variability, cortisol levels, immunological markers) limits the strength of the results. Studies should consider including breathing-based biofeedback in the VR headset to support collection of physiological data and measures of parasympathetic nervous system activity, as well as to support active self-regulation and relaxation during the MBVR. In light of the above limitations, the positive findings should be considered preliminary and more rigorous research is needed to further assess intervention efficacy.

Future research should build upon this pilot study and further assess the efficacy of MBVR among children and young adults with IBD. Most importantly, a larger RCT that includes a VR control condition and additional sources of data (e.g., biophysiological outcomes) is needed to determine intervention efficacy. Additionally, exploring longer-term use of MBVR (e.g., one month), as well as follow-up assessments (e.g., weekly, monthly), would provide important information about intervention efficacy on key IBD outcomes, practice effects, and maintenance of treatment effects. Lastly, it would be valuable to conduct RCTs of MBVR across a diverse array of settings to explore intervention feasibility and efficacy in a range of pediatric IBD medical and home settings. Future research could help answer questions related to optimal MBVR delivery and dose required to achieve short- and long-term effects among pediatric IBD populations.

In summary, this pilot study offers preliminary support for the feasibility and acceptability of MBVR among children and young adults with IBD and suggests the intervention can improve anxiety and pain in an outpatient medical setting. These findings underscore the importance of further examining MBVR and its ability to support the psychological and physical wellbeing of pediatric IBD populations.

## Figures and Tables

**Table 1:** Qualitative feedback: A selection of responses about participants’ MBVR experience and areas for future use.

MBVR Experience	Representative Quotations
Relaxation	“Really good for soothing Crohn’s.” “Felt like a new world; Relaxing scenery; Peaceful.” “Feel calmer.” “Scenery was relaxing; Loved the aurora lights.” “It was relaxing when looking at the nature and how that was incorporated into the controlled breathing with the lights.”
Enjoyment	“I wish they had this technology or other anxiety/pain management offered to me when I was a younger patient.” “I really enjoyed it, I’d like a Gear VR headset for Christmas.” “I got happy symptoms, I would use it every day.” “Very effective… I experienced mindfulness before, but it was never as effective as this.” “MBVR could help in getting used to mindfulness while my mind still wanders.” “I have an HTC vive. I liked it and might try other relaxing environments now.” “I’d recommend it to friends and other patients with IBD.”
**Future Use**	
Clinical	“Would be helpful for people in the hospital or undergoing procedures or injections (e.g., blood draws, MRI, colonoscopy, infusion).” “Would be beneficial before and after stressful procedures.” “Would be helpful before going to surgery, MRI, CT, or other procedures and if someone was afraid of needles.” “Would be good before blood draws or anything with needles.” “Would be helpful for patients at diagnosis.”
Non-clinical (e.g., daily life)	“Would be helpful before exams and matches.” “Would be helpful before work and to start the day and reduce stress.” “Would be helpful prior to tests in school.” “MBVR would be helpful for parents and siblings too.” “A take home version for patients and all family members.” “There should be a study including parents due to the overwhelming events that they go through with their child with IBD.”
User experience	“IBD kids could choose their setting (i.e., mountains, beach, forest, etc.).” “Create a more calming environment for MBVR; Turn off lights and make the room quiet to get rid of external stimuli; Use headphones.” “VR could be more interactive like involving hands to touch the waterfall or animals during mindfulness; Would be cool if you could see yourself walking in nature.”

Qualitative feedback regarding MBVR experiences was gathered post-intervention from a satisfaction survey and a brief semi-structured interview with research staff.

**Table 2:** Ratings of Feasibility and Acceptability by Age Group.

Satisfaction Questions Rated on a 1 (Poor/Not at All) to 5 (Ideal/Excellent) Scale	Mean	SD
**Participants < 18 years old**		
Enjoyment	4.43	0.80
Relaxation	4.35	0.79
Length of MBVR	3.88	1.17
Home use	4.05	1.10
**Participants** **≥ 18 years old**		
Enjoyment	4.26	1.05
Relaxation	4.37	0.96
Length of MBVR	4.26	0.99
Home use	4.21	1.08

## Data Availability

The data presented in this study are available on request from the corresponding author. The data are not publicly available due to privacy.
